# Diverticular disease of the small bowel: a rare cause of the duodenojejunal flexure obstruction (a case report)

**DOI:** 10.11604/pamj.2021.38.213.27575

**Published:** 2021-02-24

**Authors:** Arwa Guediche, Soumaya Ben Amor, Walid Mnari, Mabrouk Abdelaali, Waad Farhat, Houcem Ammar, Mohamed Amine Said, Mejda Zakhama, Wided Bouhlel, Om Keltoum Sellem, Nabil Ben Chaabene, Mondher Golli, Ali Ben Ali, Leila Safer

**Affiliations:** 1 Gastroenterology Department, Fattouma Bourguiba Hospital, Monastir, Tunisia,; 2 Radiology Department, Fattouma Bourguiba Hospital, Monastir, Tunisia,; 3 General Surgery Department, Sahloul Hospital, Sousse, Tunisia,; 4 Nutrition Department, Fattouma Bourguiba Hospital, Monastir, Tunisia

**Keywords:** Diverticula, small intestine, gastrointestinal obstruction, case report

## Abstract

The small bowel is the least common site for diverticula in the entire gastrointestinal tract. Chronic upper intestinal obstruction due to diverticula is very rare. We report a case of multiple small bowel diverticula causing mechanical obstruction of the duodenojejunal flexure.

## Introduction

The small bowel is the least common site for diverticula in the entire gastrointestinal (GI) tract, and most of them are asymptomatic. Related complications such as diverticulitis, perforation, bleeding or intestinal obstruction appear in 10-30% of the patients increasing morbidity and mortality rates [1]. We report a case of a 51-year-old female with giant compressive paraduodenal diverticulum and multiple other diverticula of the small bowel revealed by symptoms of upper gastrointestinal tract obstruction and weight loss of 20kg in just three months.

## Patient and observation

**Figure 1:**
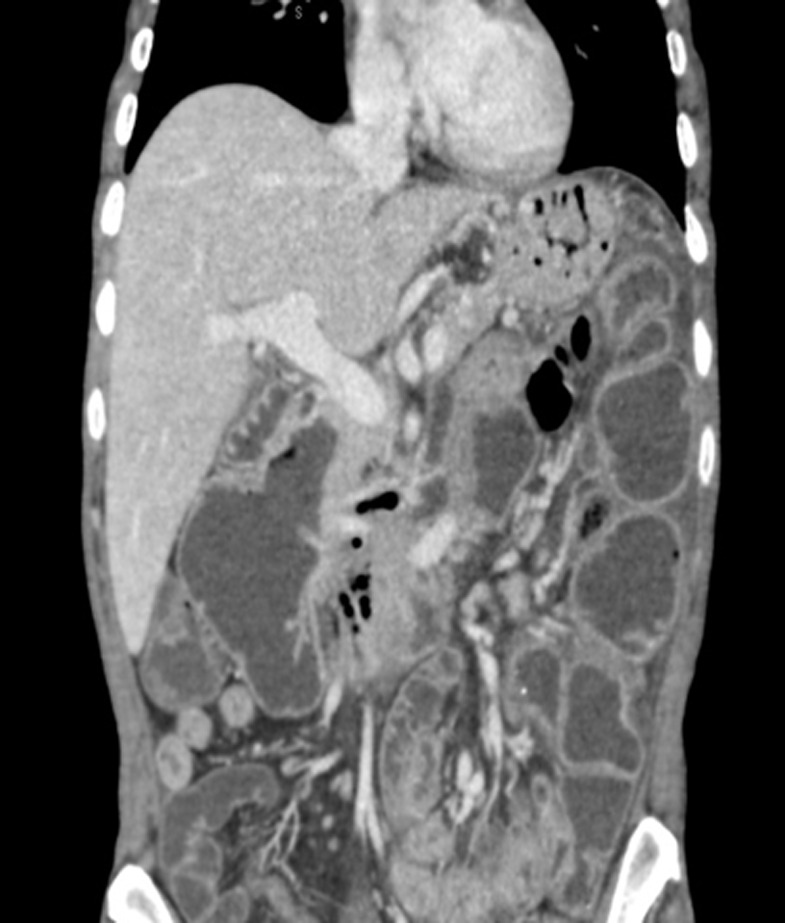
computed tomography showing distended proximal small bowel loops with multiple diverticula

**Figure 2:**
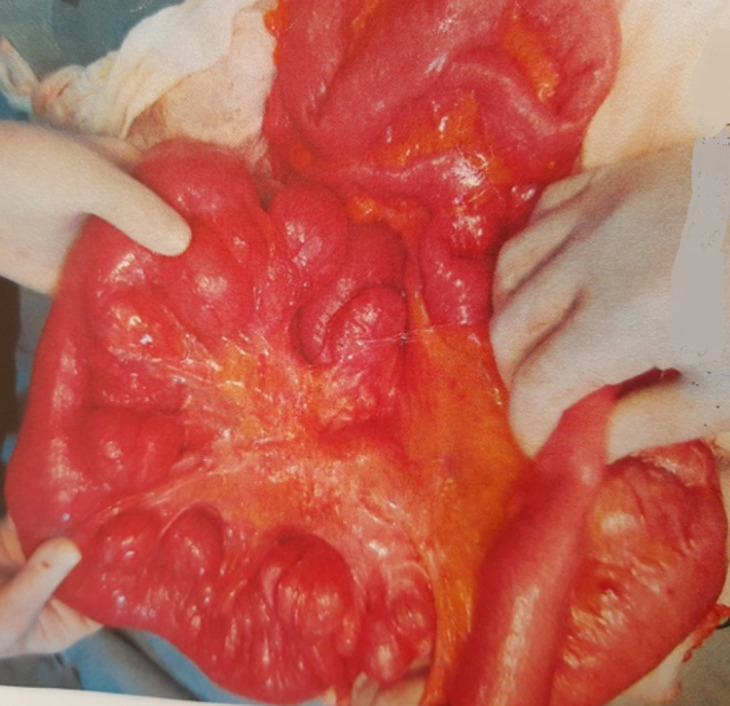
diffuse jejunal diverticula reaching duodenum during the exploratory laparotomy

A 51-year-old woman, without a medical family history, was admitted to the hepato-gastro-enterology department in late December 2016 with a 6-month history of delayed post-prandial vomiting and significant weight loss (about 20kg in three months). She was operated in July 2016 for a left crural hernia. At physical exam, her body mass index was 17.9kg/m^2^. Abdominal exam revealed an obvious fasting lapping without palpable mass or other abnormalities. Abnormal laboratory findings included anemia (haemoglobin-10.9g/dl; MCV 87fL), hypoalbuminemia (28g/l) and hypocholesterolaemia (total cholesterol 2.2mmol/l). Upper gastrointestinal endoscopy showed multiple duodenal diverticuli, a distension of the 1^st^ and 2^nd^ parts of the duodenum with stomach stasis. The abdominal computed tomography (CT) demonstrated distended proximal small bowel loops with multiple diverticula, of them one was paraduodenal, giant measured 9cm*8cm*6.5cm and exerts a compression effect over the inferior duodenal angle (Figure 1). The patient underwent laparotomy. Upon exploration, we found diffuse jejunal diverticula reaching duodenum (Figure 2). A resection of diseased segment of jejunum, the third and fourth portions of duodenum with a duodeno-jejunal latero-terminal anastomosis were carried out. A cholecystectomy was also performed. The patient´s post-operative course was uneventful and over three months; she gained up to 15kg.

## Discussion

Small bowel diverticulosis (SBD) represents an uncommon pathology [2]. The etiology of this entity remains unknown. The most widely accepted theory is that irregular intestinal contractions generate increased segmental intra-luminal pressure, favoring the formation of diverticula [3, 4]. Clinical aspects of this entity are variable. Majority of people with SBD are asymptomatic or have minor, non-specific gastro-intestinal symptoms, and found incidentally on imaging studies or surgery performed for unrelated causes [5]. Kouraklis *et al*. [6] showed the incidence of different presentations to be as follows: abdominal pain 64%, chronic obstruction 10-25%, GI bleeding 15%, malabsorption 3.5-12% and perforation 2%. Ten to 20% present with acute abdomen due to development of complications such as diverticulitis, fistula formation, GI hemorrhage, perforation and obstruction [5]. Intestinal stenosis can be caused by sizeable diverticulum which may apply pressure to the adjacent bowel wall, as was the case with our patient. In addition, repeated episodes of diverticulitis, volvulus, intussusceptions or small bowel stones may be at the origin of mechanical obstruction [7] and laboratory findings in SBD tend to be non-specific.

In our case, laboratory studies revealed malabsorption that could be justified by the nonsynchronous peristaltic movement of the bowel, the dilation of the diverticula, the stasis of the intestinal content and the bacterial overgrowth [8]. Upper GI endoscopy is an important investigation for diagnosis of duodenal diverticulosis. It is successful in diagnosing duodenal diverticulosis (DD) in more than 75% of patients [9]. The rate of failure of endoscopy to diagnose diverticular malformation may increase if it is situated in the third or fourth part of the duodenum. Apart from the bleeding, esophagogastroduodenoscopy has no place in the diagnosis of diverticular complications. In such cases, a series of plain abdominal X-ray could reveal distension of the small bowel, air-fluid levels and pneumoperitoneum. Barium follow-through studies and enteroclysis are more specific, although their utility is limited in emergency situations. CT may show focal areas of evagination on the mesenteric side of the gut, localized intestinal wall thickening, abscesses, free abdominal fluids and pneumo-peritoneum [2].

Laparoscopy is a valid diagnostic approach for complicated cases, and total laparoscopic treatment of a sizeable jejunal diverticulum was recently reported [2]. Elective surgical treatment of asymptomatic diverticulum is not justified. Laparotomy is mandatory in cases of perforation, abscesses or obstruction. The treatment of choice for SBD, often performed emergently is resection of the diseased part, in order to avoid further complications [7]; but, if diverticula involves a long intestinal segment, resection should be limited to the perforated, stenotic or inflamed part in order to avoid short bowel syndrome [2].

## Conclusion

Although small bowel diverticula are uncommon, we should think about them as a possible cause of upper gastrointestinal obstruction and malnutrition.
